# Monte Carlo simulations using PELE to identify a protein–protein inhibitor binding site and pose

**DOI:** 10.1039/d0ra01127d

**Published:** 2020-02-17

**Authors:** Lucía Díaz, Daniel Soler, Gary Tresadern, Christophe Buyck, Laura Perez-Benito, Suwipa Saen-Oon, Victor Guallar, Robert Soliva

**Affiliations:** Nostrum Biodiscovery Jordi Girona 29, Nexus II D128 08034 Barcelona Spain rsoliva@nostrumbiodiscovery.com; Computational Chemistry, Janssen Research & Development, Janssen Pharmaceutica N. V. Turnhoutseweg 30, B-2340 Beerse Belgium gtresade@its.jnj.com; Barcelona Supercomputing Center, Join IRB-BSC Program in Computational Biology Spain; ICREA Passeig Lluís Companys 23 E-08010 Barcelona Spain

## Abstract

*In silico* binding site location and pose prediction for a molecule targeted at a large protein surface is a challenging task. We report a blind test with two peptidomimetic molecules that bind the flu virus hemagglutinin (HA) surface antigen, JNJ7918 and JNJ4796 (recently disclosed in van Dongen *et al.*, *Science*, 2019, **363**). Tests with a series of conventional approaches such as rigid (receptor) docking against available X-ray crystal structures or against an ensemble of structures generated by quick methodologies (NMA, homology modeling) gave mixed results, due to the shallowness and flexibility of the binding site and the sheer size of the target. However, tests with our Monte Carlo platform PELE in two protocols involving either exploration of the whole protein surface (global exploration), or the latter followed by refinement of best solutions (local exploration) yielded remarkably good results by locating the actual binding site and generating binding modes that recovered all native contacts found in the X-ray structures. Thus, the Monte Carlo scheme of PELE seems promising as a quick methodology to overcome the challenge of identifying entirely unknown binding sites and modes for protein–protein disruptors.

## Abstract 2

PELE prospectively unveils the binding site and mode of a protein–protein disruptor.

## Graphical abstract

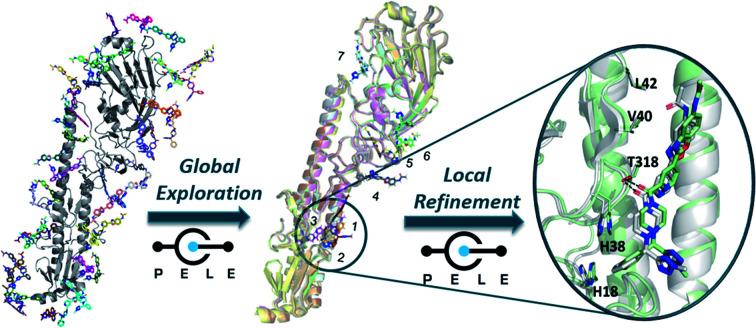

## Introduction

Extracellular protein–protein interaction (PPI) disruption is currently one of the most successful therapeutic approaches; many top selling drugs are recombinant proteins and antibodies that block extracellular protein contacts.^1^ With a success rate from first human trials to regulatory approval of around 15%, and an extensive pipeline of antibodies in clinical development many new biologic drugs are expected to launch over the coming years.^2^ Despite this, therapeutic proteins suffer from limitations (parenteral route of administration, production costs) and the requirements of some clinical indications or treatment regimes mean the discovery of small molecules as alternatives to therapeutic proteins remains a high priority. However, protein–protein interfaces are characterized by extended, flat or shallow surfaces, often dominated by polar contacts, typically not straight forward for targeting by small organic drug molecules compatible with oral route administration. Indeed, the challenges of inhibiting or modulating protein–protein interactions are well known^3,4^ and computational methods that can help with the identification of ligands, or even their likely binding sites would be of great value and potential impact.

The complexity of protein–protein interactions can be understood by considering that many proteins have evolved elongated structures whose domains are usually composed of α-helices and β-sheets placed parallel to the longest protein axis, stretching out a series of extended, narrow and shallow grooves, which are engaged by their protein partners *via* insertion of a series of side chains in a “ladder-shaped” pattern. Small molecules targeted at the surface interaction sites on such proteins must be fairly rigid and capable of inserting themselves in these grooves if they are to have good potency. However, this is not easily achieved, as the long grooves display a dynamic adaptation at different levels of the “ladder”, something that cannot be inferred from the static X-ray crystal structures. Examples of this phenomenon have been described both for extracellular proteins such as IL-2/IL-2R disruptors^5,6^ and intracellular proteins such as the androgen receptor.^7^

Drug discovery of inhibitors disrupting PPIs poses many challenges and traditional computational methods often struggle to overcome these. A recent systematic analysis revealed that PPI cavities show almost no overlap in property space with those of druggable protein ligand complexes,^8^ thus identifying chemical hit matter can be tough, and optimizing it to adhere to drug-like physicochemical properties is a further challenge.^9^ Still, classical docking approaches have been applied to find hits for PPI targets but the challenge for docking can be understood given that most scoring functions have evolved and been evaluated using available protein-ligand structure datasets which only contain a small proportion of ligands bound at PPI binding sites. Some successes have been reported but similar limitations of the available chemical molecules also apply; and obviously these methods are of no use when the binding sites maybe unknown. Alternatively, despite a few reports of success, hit/lead generation strategies such as modern fragment-based drug design also struggles for targeting large protein–protein interfaces.^3^

Qualitative computational methods have emerged as useful to identify possible binding site and interaction “hot-spots”.^10,11^ Site finding methods such as FTMap^12^ and SiteMap^13^ can provide guidance for regions where small molecules may interact, but their power to discriminate true from false sites breaks down for less druggable sites.^14^ Given the dynamic nature of protein–protein interactions,^15^ it is perhaps not surprising that methodologies based on molecular simulation are proving useful. So-called “mixed solvent MD” methods use molecular dynamics (MD) simulations performed with small co-solvent organic fragments to reveal protein surface binding sites.^16,17^ Meanwhile, sophisticated large-scale MD studies coupled with statistical modelling have been able to identify binding sites in a *de novo* fashion but at significant computational cost.^18^ It remains to be seen if these approaches can impact prospective drug discovery with the throughput and robustness to identify unknown binding sites and importantly, binding poses. In general, there are few reliable computational approaches that can be applied to PPI drug discovery. Here we demonstrate how Monte Carlo (MC) simulations performed with PELE^19^ are capable of identifying both the binding site and mode, with relative ease and *via* a simple and reproducible application protocol.

**Fig. 1:**
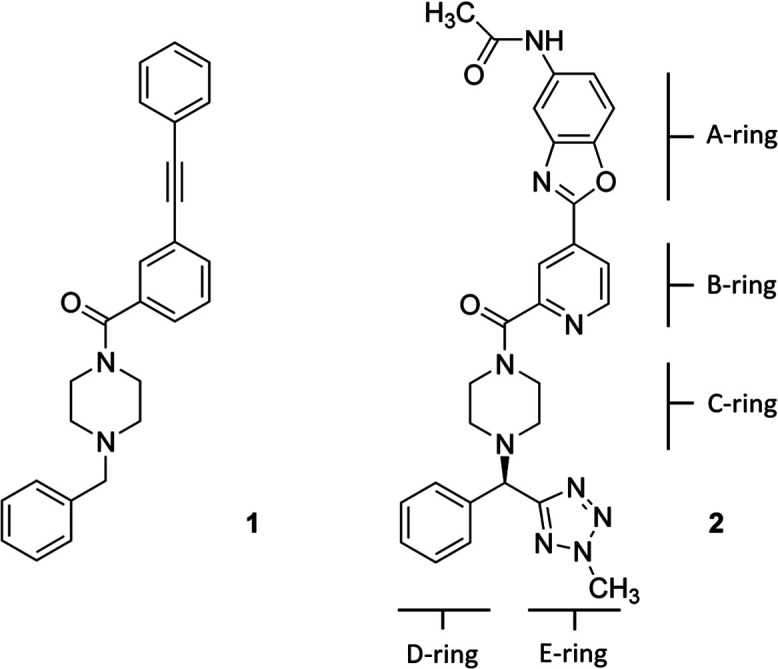
(Left) 2D structure of compound 1, JNJ7918 micromolar HTS-derived HA binder; (right) 2D structure of compound 2 JNJ4796, an optimized nanomolar HA binder (rings are tagged with letters for discussion purposes).

Hemagglutinin (HA) is one of the two proteins found on the surface of the influenza viruses. It is an antigenic glycoprotein assembled as a homo-trimer of identical subunits that are formed of two disulphide-linked polypeptides: membrane-distal HA1 and the smaller, membrane-proximal, HA2. HA is responsible for host cell binding and subsequent fusion in the endosome after the virus has been taken up by endocytosis. Therefore, its interaction with host-cell receptors is a critical step in the infectious cycle of the virus. Janssen researchers first developed potent, broadly neutralizing antibodies (bnABS) that bind the highly conserved HA stem region at the interface of HA1 and HA2.^20^ Subsequently, the study of the complementarity determining regions (CDRs) of those bnABS inspired the development of potent cyclic peptidic inhibitors^21^ whose X-ray complexes show they closely mimic the “ladder-type” interaction revealed for bnABS at the HA1/HA2 groove. Lastly, this rich structural information led to the development of small molecules targeted at the same site. Through an HTS campaign Janssen discovered micromolar compound JNJ7918 (1 in Fig. 1), which finally evolved to lead compound JNJ4796 (2, in Fig. 1).^22^ This peptidomimetic remarkably mimics bnABS and cyclic peptides and owes its potency to a series of slight, dynamic rearrangements in the “ladder-type” groove at HA1/HA2.

The goal of the present study was to test the performance of a series of relatively efficient approaches that can be employed to determine the binding site and binding mode for precursor JNJ7918 and its derived lead JNJ4796 on the whole surface of HA. The work was designed considering limited time and resources typical of some drug discovery projects and therefore slower, compute intensive techniques such as molecular dynamics were not considered. We found that the most simplistic approaches such as docking or ensemble docking with several techniques did not, for the most part, yield useful models. However, a variety of protocols based on the Monte Carlo program PELE consistently delivered good results in simulations whilst taking only a few hours on a modest compute cluster. Of note, at the time of the test, the work was performed in a blind fashion as the HA-JNJ4796 complexes (entries 6CF7, 6CFG) were not available in the protein data bank and not provided to scientists at Nostrum Biodiscovery.

## Methodologies

### Protein system preparation

All HA structures were first pre-processed and refined with Protein Preparation Wizard of Maestro.^23^ This pipeline is designed to correct most deficiencies found in protein data bank structures such as missing side chains and loops, double occupancies, missing hydrogen atoms, titration of ionizable residues, flipping of wrongly assigned Asn and Gln side chains, *etc.* The resulting structures were minimized using default parameters and visually inspected for a final quality control.

### Docking calculations

All docking calculations on the HA structures were carried out with Schrodinger's Glide 2019-2 using its Standard Precision (SP) scoring function.^24^ In cases where SP failed to give a productive pose, the Extra Precision (XP)^25^ scoring function was used, although in general results were found to be similar to SP. The peptidomimetic molecules were first processed with Schrodinger's Ligprep (protonation state at pH 7.4 ± 0.5), to generate 3D energy-minimized molecular structures with correct tautomeric and ionization states. ESP charges were obtained with Jaguar single-point calculation at M06/6-31G** level of theory.^26^ Of note, the B and C-rings (pyridine and piperidine) were predicted to be neutral. The grid was customized to include the entire protein in docking attempts to locate binding sites. Results were assessed by examining the best 10 energy poses in terms of the docking glide-score.

### Ensemble generation *via* normal mode analysis (NMA)

A conformational ensemble of 10 models of the HA receptor was generated *via* PELE by means of carbon alpha normal mode analysis (NMA) with a subsequent all atom minimization.^27^ To decide the direction of the movement a contribution of 75% came from the main mode and 25% from the rest. The main mode was randomly selected from the first six modes at each PELE step with amplitude of the movement of 1.25 Å.

### Ensemble generation *via* MODELLER

Version 9.22 of MODELLER^28^ was used to obtain an ensemble of 10 structural models of the HA receptor. The crystal structure of hemagglutinin in complex to a cyclic peptide (protein data bank (PDB)^29^ id: 5W6T)^21^ was used as a template for the generation of the conformational ensemble using the standard protocol implemented in MODELLER. The quality of the models was assessed by means of MODELLER's internal scoring function, the Discrete Optimized Protein Energy (DOPE).^30^ All the models were subjected to the same protocol described in the protein system preparation section prior to the docking calculations.

### PELE algorithm

PELE^19^ is a two-stage MC algorithm comprising: a first perturbation stage, where the ligand is randomly translated and/or rotated and the protein is perturbed using a normal mode method based on an anisotropic network model (ANM) or on a principal component analysis (PCA) of a set of diverse structures.^31^ A second stage follows, where the structure is rebuilt with a side chain rotamer prediction followed by a minimization with varying degrees of constraints on alpha carbons and the ligand centre of mass. The resulting proposal is accepted or rejected based on a Metropolis criterion, after which the whole cycle is carried out iteratively. This basic algorithm has been applied to the study of multiple drug discovery^31–33^ and protein engineering problems.^34,35^

For the present work, the adaptive version of the algorithm was applied. Adaptive PELE is composed of three main steps: sampling, clustering, and spawning, which are run iteratively.^36^ In the sampling phase a series of independent trajectories are run (typically from a few dozen up to thousands). These trajectories are generated with the classical PELE approach described above. We use rounds (epochs) of N simulations (trajectories) of length *M*, each one running on a computing core (using an MPI implementation). The clustering phase then cluster all conformations generated in all previous epochs. A number of approaches can be implemented. Typically, we use ligand RMSD as a metric for clustering. Each cluster has a central conformation and a similarity RMSD threshold, so that a structure belongs to that cluster if its RMSD with the central conformation is smaller than the threshold. When a structure does not belong to any cluster, a new one is created, defining a new cluster centre. In the clustering process, the maximum number of comparisons is *k* × *n*, where *k* is the number of clusters, and *n* is the number of explored conformations in the current epoch. The ruggedness of the energy landscape sets the most suitable RMSD value. The more complex the energy landscape is, the lower the RMSD thresholds should be to ensure a proper discretization in regions that are difficult to sample. Finally, the spawning phase chooses the seeds to be used for the next iteration (next epoch). By stopping simulations and adaptively spawning them with new initial structures for the next iteration, we bypass the problem of getting trapped in local minima. This effectively improves the search in poorly sampled regions. The selection strategy can also be used for biasing the sampling to interesting areas based on user knowledge of the system. A series of reward functions can be implemented, fine-tuning the degree of bias (the default protocol rewards poorly sampled regions).

In the present work, two different PELE protocols were applied, depending on the result to be achieved, namely:

### PELE global exploration

**Fig. 2:**
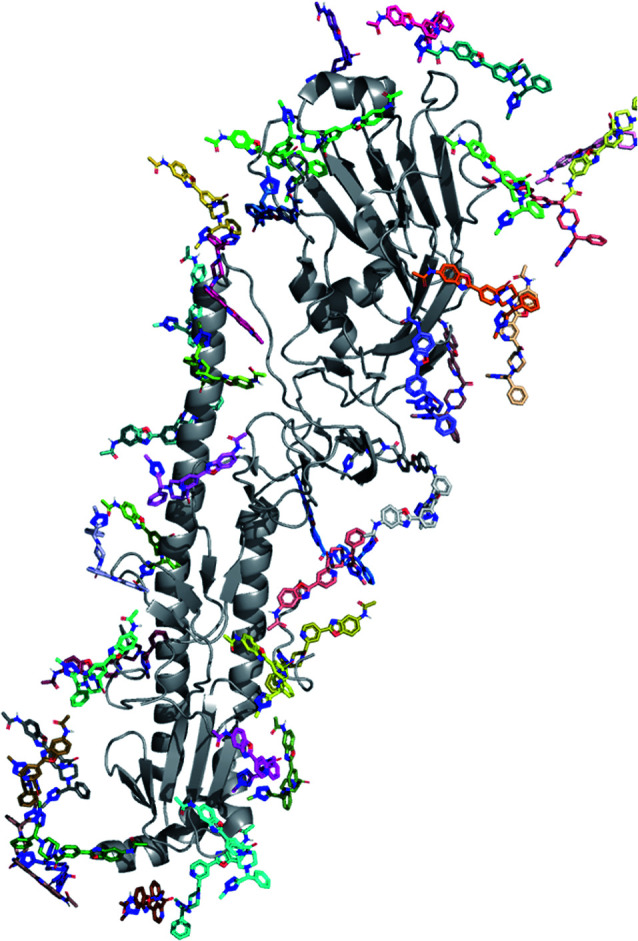
The 40 initial ligand positions placed randomly around the entire protein surface.

This protocol performs a dynamic exploration of the whole surface with *N* (initial) copies placed around the entire protein. In the case of hemagglutinin, 40 initial PDB files where the ligand was placed at random positions around the entire protein surface were first generated (Fig. 2). These files were uniformly distributed among 249 processors and used as input to launch a multiprocessor PELE simulation. Since the binding site was assumed to be unknown, large rotations and translations were applied to the ligand. The simulation was run for a total of 400 MC steps for both compound 1 and 2 (totalling 9 and 12 hours of simulation each per ligand); results were analysed by inspecting the lowest interaction energy poses.

### PELE local refinement

In the case of compound 2, the lowest interaction energy poses found in the global exploration were used as input for a local refinement. Low and medium range rotations and translations were applied to the ligand in this case. In addition, the ligand was only allowed to explore the area around each of the binding sites found in the previous global exploration. The simulation was run for 1 hour on 175 processors.

In all calculations, PELE used the all atom OPLS2005 force field^37^ with an OBC implicit solvent model. Parameters for the ligand were obtained by the following protocol. The charges were derived from RESP methodology, calculated at M06/6-31G** level of theory using Jaguar. The force field parameters were transferred from OPLS2005. PELE energy profiles presented in the results section are the interaction energy plot against distance to the centre of mass (COM) of Thr318 (one of the key residues anchoring compound 1). Interaction energies are defined by *E*(AB) − *E*(A) − *E*(B), where AB stands for the complex, A for the receptor and B the ligand.

## Results and discussion

The study was performed blind, before the public release of the crystal structures, and aimed at testing whether the different techniques could find the binding site/mode of compounds 1 and 2 assuming they could bind anywhere on the surface of hemagglutinin.

### Global docking on available X-ray structures, NMA-generated and HM-generated ensembles

Docking of both compounds was attempted first on PDB entries 5W6I, 5W6T and 5W6U (hemagglutinin–cyclic peptide complexes), making sure the grid engulfed the whole protein structure. Results were disappointing as not a single generated pose for either could reproduce a native-like pose. Inspection of the X-ray structures reveals the grooves along the longest protein axis are fairly rigid, but undergo slight rearrangements precluding binding of neither of the peptidomimetics. Clearly, receptor plasticity must be taken into account even in protein–protein contact surfaces not involved in major rearrangements.

The rigid (receptor) docking results prompted us to investigate whether a productive pose could be found by first generating conformational ensembles for hemagglutinin, followed by docking both molecules on every ensemble conformer. Two short methodologies were chosen: normal mode analysis (NMA) and homology modelling (HM).

An NMA-generated ensemble with 10 conformers was built based on PDB entry 5W6T (HA bound to a cyclic peptide). All docking grids engulfed the whole protein structure. No binding mode close to the known binding site (Thr318) was found for compound 1. Additionally, only two out of all generated poses for compound 2 were close to the X-ray pose (as in 6CF7); the best in terms of RMSD being scored as the 5^th^ best pose out of 100, reproducing the key hydrogen bond between the carbonyl oxygen of JNJ4796 and the hydrogen bond donor of Thr318 and yielding a native like conformation with 2.63 Å heavy atom RMSD with respect to the X-ray.

In addition, an HM-generated ensemble with 10 different conformers based on PDB entry 5W6T as template was also built. For compound 1, only one out of the 100 generated poses was close to the actual binding site (ranked as top 1), reproducing the key hydrogen bond to Thr318. However, for compound 2, no native poses could be found.

Therefore, rigid (receptor) docking failed at generating useful models when performed on X-ray crystal structures, whereas ensemble rigid (receptor) docking only gave mixed results: near native poses could be found for compound 1 in the case of HM-generated ensembles, and for compound 2 in the case of NMA-ensemble. Furthermore, the scoring itself was not accurate enough, as it is doubtful that the 5^th^ ranking pose for compound 2 would have been selected in a prospective application scenario, assuming the “correct” final pose was not known. Probably, the ensembles built were not rich enough to properly capture the induce fit needed to adequately bind both compounds under study. Thus, in order to efficiently capture the dynamic rearrangements in the “ladder-type” groove at the HA1/HA2 interface in the presence of either compound, a series of PELE simulations were run.

### Global PELE exploration for compound 1

**Fig. 3:**
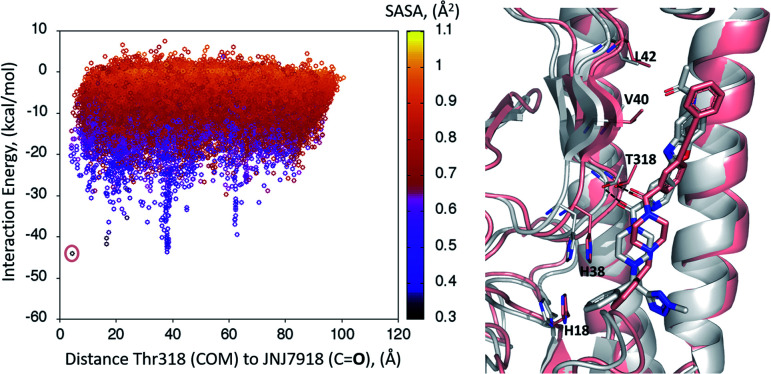
Interaction energy plot (left) against distance to Thr318 (one of the key residues anchoring compound 1). The superimposition of the lowest energy pose 1 (in salmon) with the crystal structure of related compound 26CF7 (in grey) is pictured on the right hand side image.

A first dynamic global exploration of the whole HA surface was designed. As in docking simulations, we used the HA structure found in PDB entry 5W6T, where HA is bound to a cyclic peptide. The interaction energy profile *vs.* distance to Thr318 for the global exploration of compound 1 is presented in Fig. 3, revealing four minima at different distances along the *X*-axis. Although we present the results for ease of interpretation sorted by their distance to Thr318 in the final binding site, it is important to note that if an alternative surface amino acid had been chosen it would not change the conclusions. In other words, four distinct minima exist, and their energies and identification was not dependent on the Thr318 distance. Notably, the pose of compound 1 with lowest energy (circled in salmon) closely overlaps with the binding mode of compound 2 found in the crystal structure (PDB: 6CF7), as can be seen in Fig. 3. As no crystal structure of compound 1 in complex to HA has been released to date, we present here a presumed binding mode obtained by PELE that is in line with the current reported data for this family of inhibitors.

### Global PELE exploration for compound 2

**Fig. 4:**
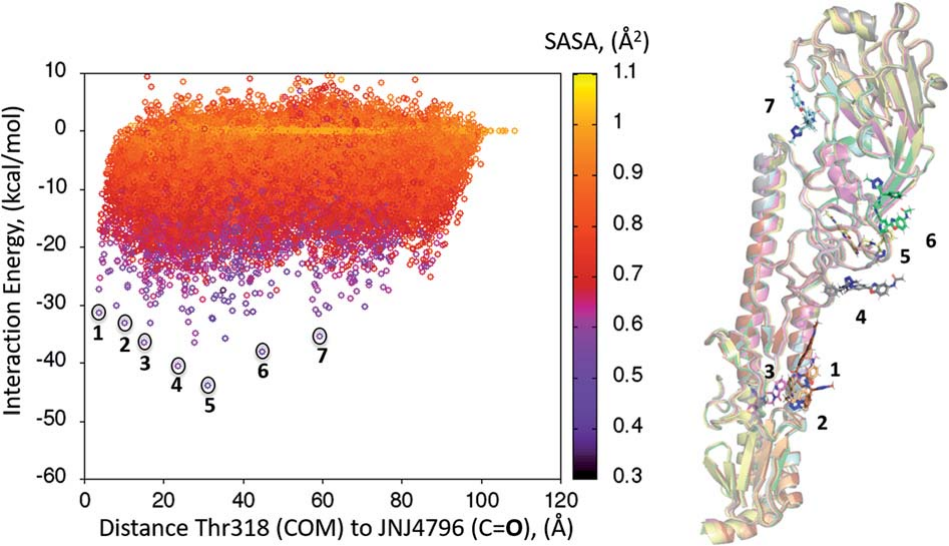
Interaction energy plot (left) against distance to Thr318 (one of the key residues anchoring compound 1). The lowest energy poses circled, whose positions on the HA surface are depicted on the right hand side image, where taken as starting structures for the second (refinement) simulation step.

A dynamic PELE global exploration for the lead compound was also attempted on PDB entry 5W6T. The energy profile for the exhaustive global exploration is found in Fig. 4. It reveals the exploration generates 7 minima, one of which (point 1) is in the vicinity (*ca.* 2 Å) of Thr318, the key H-bonding anchor of compound 1. Thus, the initial exploration locates the actual binding site as one of the probable hotspots on the surface of HA. However, this initial global calculation does not generate a right binding mode. This might be due to the higher structural complexity of the lead compound as compared to its HTS precursor.

**Fig. 5:**
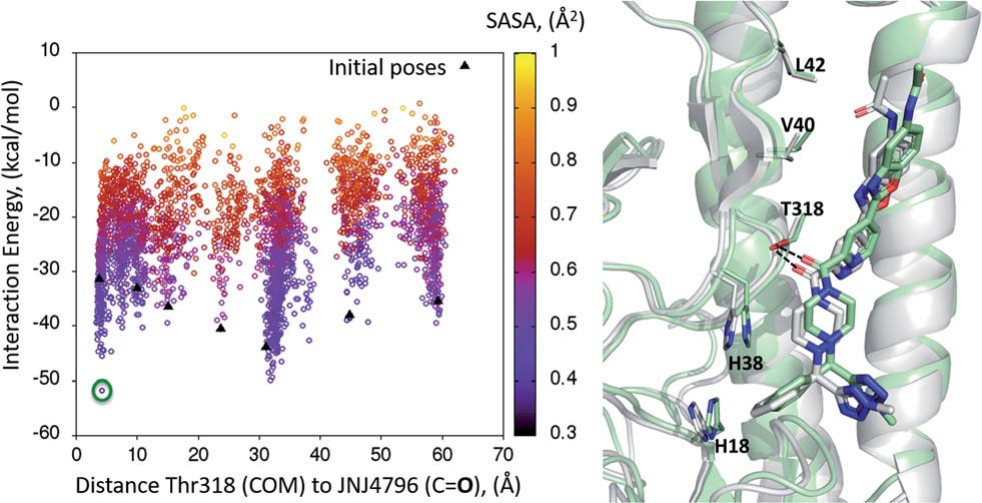
(Left) Interaction energy plot against distance to Thr318 for the PELE simulations starting with the 7 poses generated by the global exploration (Fig. 4); (right) the lowest interaction energy pose (green circle on the left plot) out of the second refinement simulation can be seen superimposed with the actual binding mode as subsequently published in van Dongen *et al.*^22^ and found in PDB entry 6CF7 (grey). The overlap with the experimental binding mode shows how the model captures the H-bond to Thr318, as well as the CH–π bonds of compound 2 with the residues lining the shallow groove.

The 7 minima highlighted in Fig. 4, which are far apart from one another, were then subjected to a second round of PELE simulations, now in local exploration mode (details on the simulation parameters can be found in the Methodologies section). The energy profile for the refinement of the 7 poses highlighted in Fig. 4 can be found in Fig. 5. Remarkably, it shows the pose that H-bonds to Thr318 as the one with the lowest interaction energy (−51 kcal mol^−1^). The lowest energy minimum circled in green Fig. 5 (left) is also seen in Fig. 5 (right). It overlaps with the binding mode disclosed in PDB entries 6CF7 and 6CFG (RMSD 1.47 Å). In fact, a detailed inspection of the predicted binding mode reveals most of the critical contacts responsible for the nanomolar activity of compound 2 are recovered in our model. The A ring (benzoxazole) is placed in the small hydrophobic cavity lined by Val40, Leu42 and Val52, Asn53 and Ile56, its amide function partly solvent exposed, with its terminal methyl group contacting Ser291 and Leu292. The critical CH–π contact with Val52 is also recovered. The B ring (pyridine) is placed between Thr49 and Thr318, the latter H-bonding directly to the carbonyl linker connecting rings B and C, as is found in the X-ray. The C–E rings are seen in the experimental structure to engage in CH–π contacts with His18, His38, Trp21 and Ile45.

Thus, an exhaustive exploration of the surface of HA followed by a refinement simulation yields a model that is at a remarkable 1.47 Å RMSD with respect to the experimentally determined structure. This test places PELE as an efficient and effective approach to not only navigate the whole surface of a protein in search of dynamic hotspots but also to predict the actual binding mode of a small molecule protein–protein disruptor.

Since the first disclosure of the PELE software,^19^ the platform has undergone significant methodological improvements. A big upgrade was achieved when the original Fortran code was ported to C++ and the MPI parallelization was optimized with Paraver software to avoid overhead and maximize job performance.^38^ Along the years, more features have been developed to address specific problems that broadened PELE's applicability domain. For instance, adding PCA components to PELE to account for non-harmonic movements,^31^ a feature to perturb explicit water molecules in the MC algorithm,^39^ the adaptive PELE version (explained above) to enhance sampling and minimize the computational demand^36^ and a series of changes to make the code more flexible in term of input/output formats which minimize user storage needs. Early reports involving the study of cytochrome P450s, myoglobin or fatty acid binding protein^19,40^ have now been complemented with multiple recent studies involving kinases, GPCRs, HIV-1 protease, epoxide hydrolase and various nuclear hormone receptors.^31,32,36,41–46^ Thus the application of PELE as a reliable induced-fit binding tool has been amply proven. We show here for the first time, the application of PELE in the context of protein–protein disruptor design, where many *in silico* methodologies struggle.

## Conclusions

The correct prediction of binding sites and modes for small molecule protein–protein inhibitors is still challenging, even in cases where no major rearrangements in the protein structure take place, as the grooves involved in binding are highly dynamic, extensive and mostly featureless. A series of computationally efficient approaches have been tested for the correct prediction of two peptidomimetic molecules that bind the surface of hemagglutinin, a 380 dalton MW HTS micromolar hit and its evolved derivative, the 566 dalton nanomolar lead compound JNJ4796.

Our tests revealed that rigid (receptor) docking could not locate the binding site nor mode. Rigid (receptor) could yield near-native poses when performed on ensembles of structures built with approaches such as NMA or homology modelling, but results were not amongst the best predicted or consistent and would have been difficult to identify in a prospective manner. However, the Monte Carlo platform PELE did find the experimental binding mode for compound 2 (and probably 1) in relatively quick simulations performed on a small compute cluster. Our results suggest PELE is a promising tool for the study of challenging protein–protein disruptors, one of the next frontiers in small molecule drug discovery.^47–49^

## Conflicts of interest

There are no conflicts to declare.

## Supplementary Material
